# Implementation of intermittent theta burst stimulation compared to conventional repetitive transcranial magnetic stimulation in patients with treatment resistant depression: A cost analysis

**DOI:** 10.1371/journal.pone.0222546

**Published:** 2019-09-12

**Authors:** Andrew B. Mendlowitz, Alaa Shanbour, Jonathan Downar, Fidel Vila-Rodriguez, Zafiris J. Daskalakis, Wanrudee Isaranuwatchai, Daniel M. Blumberger

**Affiliations:** 1 Temerty Centre for Therapeutic Brain Intervention at the Centre for Addiction and Mental Health, Toronto, ON, Canada; 2 Institute for Health Policy Management and Evaluation, University of Toronto, Toronto, ON, Canada; 3 Toronto Health Economics and Technology Assessment Collaborative, Toronto, ON, Canada; 4 Department of Psychiatry and Institute of Medical Science, Faculty of Medicine, University of Toronto, Toronto, ON, Canada; 5 MRI-Guided rTMS Clinic and Krembil Research Institute, University Health Network, Toronto, ON, Canada; 6 Non-Invasive Neurostimulation Therapies (NINET) Laboratory, University of British Columbia Hospital, Vancouver, BC, Canada; 7 Department of Psychiatry, University of British Columbia, Vancouver, BC, Canada; 8 The Centre for Excellence in Economic Analysis Research, Li Ka Shing Knowledge Institute, St. Michael's Hospital, Toronto, ON, Canada; Shanghai Mental Health Center, CHINA

## Abstract

**Background:**

Repetitive transcranial magnetic stimulation (rTMS) is an evidence-based treatment for depression that is increasingly implemented in healthcare systems across the world. A new form of rTMS called intermittent theta burst stimulation (iTBS) can be delivered in 3 min and has demonstrated comparable effectiveness to the conventional 37.5 min 10Hz rTMS protocol in patients with depression.

**Objectives:**

To compare the direct treatment costs per course and per remission for iTBS compared to 10Hz rTMS treatment in depression.

**Methods:**

We conducted a cost analysis from a healthcare system perspective using patient-level data from a large randomized non-inferiority trial (THREE-D). Depressed adults 18 to 65 received either 10Hz rTMS or iTBS treatment. Treatment costs were calculated using direct healthcare costs associated with equipment, coils, physician assessments and technician time over the course of treatment. Cost per remission was estimated using the proportion of patients achieving remission following treatment. Deterministic sensitivity analyses and non-parametric bootstrapping was used to estimate uncertainty.

**Results:**

From a healthcare system perspective, the average cost per patient was USD$1,108 (SD 166) for a course of iTBS and $1,844 (SD 304) for 10Hz rTMS, with an incremental net savings of $735 (95% CI 688 to 783). The average cost per remission was $3,695 (SD 552) for iTBS and $6,146 (SD 1,015) for 10Hz rTMS, with an average incremental net savings of $2,451 (95% CI 2,293 to 2,610).

**Conclusions:**

The shorter session durations and treatment capacity increase associated with 3 min iTBS translate into significant cost-savings per patient and per remission when compared to 10Hz rTMS.

## Introduction

Major depressive disorder (MDD) is a debilitating mental illness, accounting for 4.3% of the global burden of disease.[1] Since 2017, MDD has been identified as the leading cause of disability worldwide.[2] Although a variety of treatments are available, including both pharmacological and non-pharmacological therapies, studies have demonstrated that many MDD patients fail to achieve remission even on adequate dosages of antidepressant medication.[3–5] In the landmark STAR*D trial of sequential pharmacotherapy in MDD, the prevalence of treatment-resistant depression (TRD) was 30%, using a criterion of failure to achieve remission after two successive antidepressant treatment regimens.[6,7] The prevalence of TRD has been estimated at ~2% for the population[8] and has been associated with a contribution of 30 to 50% of the total treatment cost for depression.[9,10]

Historically, electroconvulsive therapy (ECT) was the mainstay therapy for severe TRD, and is still the most effective treatment for severe and resistant depression.[11] However, the requirement for general anesthesia, and a monitored bed during seizure induction and recovery impose significant logistical and cost constraints on system-wide ECT capacity; moreover, perceived stigma, cognitive adverse effects, and episodic memory impairment limit patient acceptability for ECT.[11] Consequently, fewer than one percent of TRD patients receive ECT and the cost and logistical demands of ECT limit the scalability of this treatment as a viable option to make meaningful reductions in the population prevalence of TRD overall.[12,13] More scalable interventions for TRD are therefore needed.

Since the introduction of TMS by Barker and colleagues in 1985 and the subsequent initial report in depression,[14,15] numerous large-scale multicentre trials and meta-analyses across thousands of patients have confirmed the efficacy and safety of rTMS in TRD.[16]. rTMS applies powerful, focused magnetic field pulses, via an inductor coil positioned over the scalp, to induce durable changes in the activity of target brain regions associated with MDD.[17] rTMS has since been widely adopted into clinical practice across the United States (US) as an effective, less invasive, and less expensive brain-stimulation intervention for TRD.[18]

Current translational research in rTMS seeks to further improve the efficacy, cost, accessibility, and time required to achieve remission from TRD. The original (2008) FDA-approved rTMS protocol, still widely used, applied 3,000 pulses of 10Hz stimulation to the left dorsolateral prefrontal cortex (DLPFC) over 37.5 min.[19,20] However, these long session durations constrain both daily treatment capacity and per session costs, limiting the widespread adoption of 10Hz rTMS.[12,21] Subsequently, a newer 3 min protocol called intermittent theta-burst stimulation (iTBS) achieved comparable or superior physiological potency to longer conventional protocols in preclinical studies,[22,23] and showed antidepressant efficacy in preliminary sham-controlled clinical trials.[24,25] Recently, the THREE-D trial was published as the first randomized non-inferiority trial directly comparing 37.5 min 10Hz rTMS to 3 min iTBS for the left DLPFC in TRD.[21] iTBS proved non-inferior to 10Hz rTMS in reducing depression scores on both the Hamilton Rating Scale for Depression (HRSD-17) and the self-report Quick Inventory of Depressive Symptoms (QIDS-SR).[21] Response and remission rates were also non-inferior for iTBS, even though the iTBS protocol required less than a tenth of the time to administer compared to conventional 10Hz rTMS.[21]

In August 2018, the US Food and Drug Administration (FDA) approved iTBS for treatment of adults with TRD.[26] This decision was based on supporting clinical evidence from the THREE-D trial, citing a similar side-effect, safety, and tolerability profile without compromising the effectiveness of treatment.[26] Given the evidence that iTBS is non-inferiority to 10Hz rTMS, further analysis is now needed to provide estimates of the potential economic impact of implementing iTBS in clinical practice. The aim of this study was to compare the direct treatment costs per course and per remission for iTBS versus conventional 10Hz rTMS protocols in patients with TRD.

## Materials and methods

### Study design

The study was approved by the Centre for Addiction and Mental Health Research Ethics Board. Protocol 179–2012. Written informed consent was obtained. We performed a cost analysis to compare the per course and per remission costs following a course of either iTBS or 10Hz rTMS using patient-level data from the completed THREE-D trial.[21]

### Participants

From September 3, 2013 to October 3, 2016, a total of 385 patients participated and were included in the primary analysis of the THREE-D trial.[21] Participants included adults age 18–65 with a Mini-International Neuropsychiatric Interview-confirmed diagnosis of diagnosis of MDD who did not respond to or could not tolerate adequate pharmacotherapy.[21] Patients were recruited from referrals to neurostimulation centres at three Canadian university hospitals. Patients were excluded if they declined to participate or met the exclusion criteria of the trial, including a past history of substance abuse, psychotic disorders, central neurological illness (including epilepsy), or any rTMS contraindications.[21] Eligible patients were randomized to receive either 10Hz rTMS or iTBS of the left DLPFC. Written informed consent was obtained from all study participants. All participants who were able to complete a minimum of four weeks corresponding to a minimum adequate course of treatment were included in this analysis. Further details on participant recruitment for the THREE-D trial can be found elsewhere.[21]

### Perspective and time horizon

A costing analysis was undertaken from the perspective of the healthcare system and included costs associated with physician assessments, technician time, and treatment equipment. As participants in both study groups completed four to six weeks of once-daily weekday treatment sessions,[21] The time horizon of the study was the duration of the complete course of treatment per patient following initial assessment.

### Variables

Costing was performed at the patient-level. Data obtained from the THREE-D trial included the number of treatment sessions per patient.[21] Costs were obtained from published literature, expert opinion, and equipment manufacturers. Unit prices for each cost item are summarized below and in Table 1. All costs are reported in 2018 United States dollars (USD). Data on adverse events were not included in the cost analysis as the THREE-D trial showed that self-reported adverse events and serious adverse events occurred at low rates that did not significantly differ between study groups.[21]

**Table 1:** Cost and controlled treatment parameters.

		10Hz rTMS		iTBS		
Parameter	Unit	Base Case	Range	Base Case	Range	Source
**Controlled Treatment Characteristics**						
Length of Session	Minutes per session	45	(30–60)	15	(10–30)	Expert opinion
Equipment capacity	Treatment sessions per day	7	(6–8)	20	(15–30)	Expert opinion
Remission rate^†^ (%)	Rate of remission per course of treatment	30	(20–40)	30	(20–40)	From THREE-D trial [21]
Core equipment amortization period	Annual period	5	(3–10)	5	(3–10)	Expert opinion
Coil amortization period	Annual period	1	(1–5)	5	(1–5)	Expert opinion
**Cost Parameters ($)**						
Core Equipment^‡^	Core equipment package cost	50,000	(37,500–62,500)	73,000	(54,750–91,250)	Manufacturer suggested
Maintenance	Annual maintenance cost	2,500	(1,875–7,500)	2,500	(1,875–3,125)	Expert opinion
Coil^‡^	Cost of coil	19,000	(14,250–23,750)	19,000	(14,250–23,750)	Manufacturer Suggested
Technician Services	Wage (Cost per hour)	30	(20–50)	30	(20–40)	Expert opinion
Initial Physician Assessment	Cost per assessment	160	(100–500)	160	(100–500)	Medicare and Medicaid Schedule HCPS 90792 [27]
Ongoing physician assessments^§^	Cost per assessment	120	(100–300)	120	(100–300)	Medicare and Medicaid Schedule HCPS 90838 [27]

Note: Costs are in 2018 United States dollars (USD)

^†^Remission rate was defined as patients achieving HRSD-17 scores <8 indicating a lessening of depressive symptoms following treatment. The baseline remission rate of 30% was derived from the average proportion of patients achieving remission following either 10Hz rTMS or iTBS treatment.

^‡^Range was obtained from ± 25% of the reference value

^§^Assessments were assumed to be once per week of treatment and range was obtained through expert opinion

HCPS indicates healthcare common procedure coding system; HRSD, Hamilton Rating Scale for Depression; iTBS, intermittent theta burst stimulation; rTMS, repetitive transcranial magnetic stimulation.

### Equipment capacity

Assuming a typical eight-hour workday with a one-hour lunch break, and a session duration of 45 minutes for conventional 10Hz rTMS (including setup time plus 37.5 min treatment time) and 15 minutes for iTBS (including setup time plus 3 min treatment time), measures of equipment capacity were estimated to be an average of 20 sessions per day for iTBS and seven per day for 10Hz rTMS. These daily treatment capacity estimates were then used in conjunction with staff, overhead, and equipment costs to estimate per session costs for iTBS and 10Hz rTMS.

### Equipment and coil costs

Costs associated with the rTMS device and associated electromagnetic coil were obtained directly from device manufacturers. Core equipment costs included costs associated with the stimulator, cart, arm, isolation transformer, and basic cooling system (if necessary). Base iTBS system costs included a high-performance cooling system necessary to perform multiple repeated iTBS sessions consecutively without the need to change coils due to overheating. For the baseline analysis, a five-year amortization period was assumed for core equipment based on standard medical equipment maintenance and replacement guidelines.[28] To derive the cost of core equipment per course of treatment, an annual equipment cost was first estimated from the base equipment cost and amortization period. Equipment capacity estimates and the assumption of 261 weekdays per year was then used to calculate a per session cost. The session cost was then multiplied by each patient’s number of treatment sessions to derive a measure of equipment cost per course of treatment for each patient.

Coil costs were obtained directly from device manufacturers. Amortization periods were assumed to differ between 10Hz rTMS and iTBS based on expert opinion and published literature indicating that the usage of iTBS requires fewer pulses and shorter durations of stimulation (600 pulses and 3 min per session) when compared to 10Hz rTMS (3000 pulses and 37.5 min per session).[29] Coil cost parameters were applied to the THREE-D trial data to estimate a coil cost per course of treatment for each patient. The coil amortization period was varied from one to five years to assess its impact on the robustness of results. In addition, an annual estimate of miscellaneous equipment maintenance costs was obtained from expert opinion to account for upkeep of the coil and core equipment. A maintenance cost per course of treatment for each patient was derived using equipment capacity and each patient’s number of treatment sessions.

### Physician assessments

Physician assessment costs (Table 1) were obtained from Medicare reimbursement rates for diagnostic psychiatric evaluations and individual psychiatric care evaluations. Each patient was assumed to receive an initial psychiatric evaluation corresponding to the Healthcare Common Procedure Coding System (HCPCS) code 90792 and subsequent weekly assessments over the course of treatment corresponding to the HCPCS code 90838.[27] Ranges for physician fees were obtained from expert opinion based on the likely length of session and type of insurance coverage. As both treatments required once-daily weekday sessions, the number of subsequent weekly assessments was calculated by dividing each patients total amount of treatment sessions by five.

### Technician wage and length of treatment session

Technician salary estimates (Table 1) were obtained from expert opinion. Technician hourly wage and the average duration of a treatment session (including setup time) were used to estimate an average measure for the cost of technician time per session. This value was then extrapolated over the number of treatment sessions for each patient to derive a cost of technician time per course of treatment.

### Cost per remission

In the THREE-D trial, iTBS showed non-inferiority to 10Hz rTMS, with the proportion of patients achieving the remission (HRSD-17 score <8) reported at 32% for iTBS (*N* = 61) and 27% for 10Hz rTMS (*N* = 51) (non-inferiority margin = 10%, *p* = 0.0005).[21] Given the finding of non-inferiority for iTBS compared to 10Hz rTMS in the THREE-D trial, for this study, an overall remission rate of 30% was assumed based on the average proportion of patients achieving remission following either treatment. Cost per remission was then calculated by dividing the cost per course of treatment by the remission rate. As other published studies have reported different remission rates, a range of rates was also obtained from published literature on 10Hz rTMS in TRD,[30,31] and additional cost per remission calculations were performed across this range as a sensitivity analysis.

### Analytical approach

Baseline patient characteristics were compared between the iTBS and 10Hz rTMS groups using Pearson’s chi-squared tests and Fisher’s exact tests for categorical variables and paired t-tests for continuous parameters.

Cost outcomes in both treatment groups were reported as mean, standard deviation (SD), median, and interquartile range (IQR). Incremental cost differences, corresponding 95% confidence intervals (CI), and *P-values* were calculated using a generalized linear model (GLM) with a log link and gamma family with treatment type as the independent variable.[32] Estimates were obtained using non-parametric bootstrapping with 1,000 replications. The gamma family specification was tested and chosen based on a Modified Parks test. *P-values* less than 0.05 were considered to indicate statistical significance. All analyses were performed in Stata Statistical Software 13 (Statacorp, US) and Microsoft Excel 2016 (Microsoft, US).

Deterministic sensitivity analyses were also performed to assess the impact of parameter uncertainty on the robustness of study results. Each parameter was varied over a clinically plausible range to consider ranges of estimates for sensitivity analyses. Coil and core equipment costs were varied using ± 25% of the reference value. For each one-way sensitivity analysis, the incremental cost per course of treatment and the incremental cost per remission were calculated between treatment groups. Results of deterministic sensitivity analyses were presented using tornado diagrams for both the incremental cost per course of treatment and the incremental cost per remission.

## Results

### Demographics

Table 2 provides baseline characteristics of all THREE-D participants considered in this study. Of the 385 participants, 192 patients (49.8%) received 10Hz rTMS and 193 (50.1%) received iTBS (Table 2). Between groups, the number of patients with a history of receiving ECT from a prior episode of depression and the proportion of patients requiring rescheduled treatment sessions were significantly different. In the iTBS group, the average number of treatment sessions per patient was 26.7 (SD 4.7) sessions, versus 26.4 (SD 4.8) sessions in the 10Hz rTMS group (*p* = 0.5427).

**Table 2:** Baseline characteristics.

Parameter	10Hz rTMS (n = 192)	iTBS (n = 193)	P Value
**Demographics**			
Age, Mean (SD)	43.4 (12.1)	41.8 (10.7)	0.1645
Years educated, Mean (SD)	16.1 (3.2)	16.5 (3.1)	0.2292
Episode length, Mean (SD)	23.8 (28.7)	21.8 (24.6)	0.4910
Men, N (%)	81 (42.2%)	74 (39.4%)	0.442
Currently employed, N (%)	67 (34.9%)	76 (39.38%)	0.363
Previous ECT, N (%)	4 (2.1%)	15 (7.8%)	0.010
Receiving psychotherapy, N (%)	73 (38.0%)	80 (41.5%)	0.492
Any anxiety diagnosis, N (%)	113 (58.9%)	100 (51.8%)	0.165
**Neurostimulation Treatment Characteristics from RCT, Mean (SD)**			
Treatment sessions	26.4 (4.8)	26.7 (4.7)	0.5427
Missed treatment sessions	0.094 (0.5)	0.13 (0.8)	0.5920
Interrupted treatment sessions	0.12 (0.4)	0.063 (0.3)	0.0744
Rescheduled treatment sessions	3.04 (3.8)	2.24 (3.7)	0.0355
**Receiving Pharmacotherapy During Treatment, N (%)**			
Antidepressant	157 (81.2%)	145 (75.1%)	0.113
Antidepressant combination	44 (22.9%)	38 (19.7%)	0.439
Antidepressant augmentation	40 (20.8%)	34 (17.6%)	0.423
Antidepressant lithium	7 (3.7%)	6 (3.1%)	0.771
Benzodiazepine	65 (33.9%)	64 (33.2%)	0.885
Anticonvulsant	10 (5.21%)	5 (2.6%)	0.185
**Treatment History, N (%)**			
One failed treatment	86 (44.8%)	87 (45.1%)	0.955
Two failed treatment	56 (29.2%)	52 (26.9%)	0.627
Three failed treatment	37 (19.3%)	39 (20.2%)	0.817
Unable to tolerate two trials	13 (6.8%)	15 (7.8%)	0.705

ECT indicates electroconvulsive therapy; iTBS, intermittent theta burst stimulation; N, number of participants; rTMS, repetitive transcranial magnetic stimulation; SD, standard deviation.

### Cost per course of treatment and cost per remission

Estimated costs per course of 10Hz rTMS and iTBS are summarized in Table 3. Overall, the estimated cost per course was $1,844 (SD 304) for 10Hz rTMS, and $1,108 (SD 166) for iTBS. Between groups, cost of technician time represented the largest difference in treatment costs, accounting for a net savings of $394 (95% CI 378 to 410) per course for iTBS. Cost of technician time accounted for 32% of the cost of treatment in the 10Hz rTMS group, compared to 18% in the iTBS group. Cost of core equipment accounted for a net savings of $70 (95% CI 66 to 74) per patient favouring iTBS, while costs attributed to the coil accounted for a net savings of $255 (95% CI 248 to 262) per patient favouring iTBS. The costs associated with physician assessments were $794 (SD 115) for 10Hz rTMS, versus $801 (SD 112) for iTBS. In terms of the overall proportion of the treatment cost, physician assessments accounted for 72% of the course cost for iTBS, compared to 43% for 10Hz rTMS.

**Table 3:** Per patient results for cumulative costs and incremental costs associated with iTBS and 10Hz rTMS.

	10Hz rTMS		iTBS			
Parameter	Mean (SD)	Median (IQR)	Mean (SD)	Median (IQR)	Incremental Cost (iTBS– 10Hz) (95% CI)*	P Value*
Cost of technician time	594 (107)	675 (450–675)	200 (35)	225 (150–225)	-394 (-410 –-378)	P<0.001
Cost of core equipment	145 (26)	164 (109–164)	75 (13)	84 (56–84)	-70 (-74 –-66)	P<0.001
Cost of coil	275 (50)	312 (208–312)	19 (3)	22 (15–22)	-255 (-262 –-248)	P<0.001
Cost of maintenance	36 (7)	41 (27–41)	13 (2)	14 (10–14)	-23 (-24 –-22)	P<0.001
Cost of physician assessments	794 (115)	880 (640–880)	801 (112)	880 (640–880)	7 (-14–29)	0.510
Total cost of course of treatment	1,844 (304)	2,072 (1,435–2,072)	1,108 (166)	1,225 (870–1,225)	-735 (-783 –-688)	P<0.001
Total cost of remission^†^	6,146 (1,015)	6,907 (4,783–6,907)	3,695 (552)	4,084 (2,900–4,084)	-2,451 (-2,610 –-2,293)	P<0.001

Note: Costs are in 2018 United States dollars (USD) and rounded to the nearest dollar.

*Estimated using a generalized linear model while employing non-parametric bootstrap analysis (1,000 replications)

^†^Remission was defined as patients achieving HRSD-17 scores <8 indicating a lessening of depressive symptoms following treatment

CI indicates confidence interval; HRSD indicates Hamilton Rating Scale for Depression; iTBS, intermittent theta burst stimulation; IQR, interquartile range; rTMS, repetitive transcranial magnetic stimulation; SD, standard deviation.

The incremental costs per course and incremental costs per remission are summarized in Table 3. iTBS yielded a net savings of $735 (95% CI 688 to 783) per course delivered, compared to 10Hz rTMS. Assuming a similar remission rate of 30% in each group, iTBS yielded a net savings of $2.451 (95% CI 2,293 to 2,610) per remission achieved, compared to 10Hz rTMS.

### Sensitivity analysis

**Fig 1:**
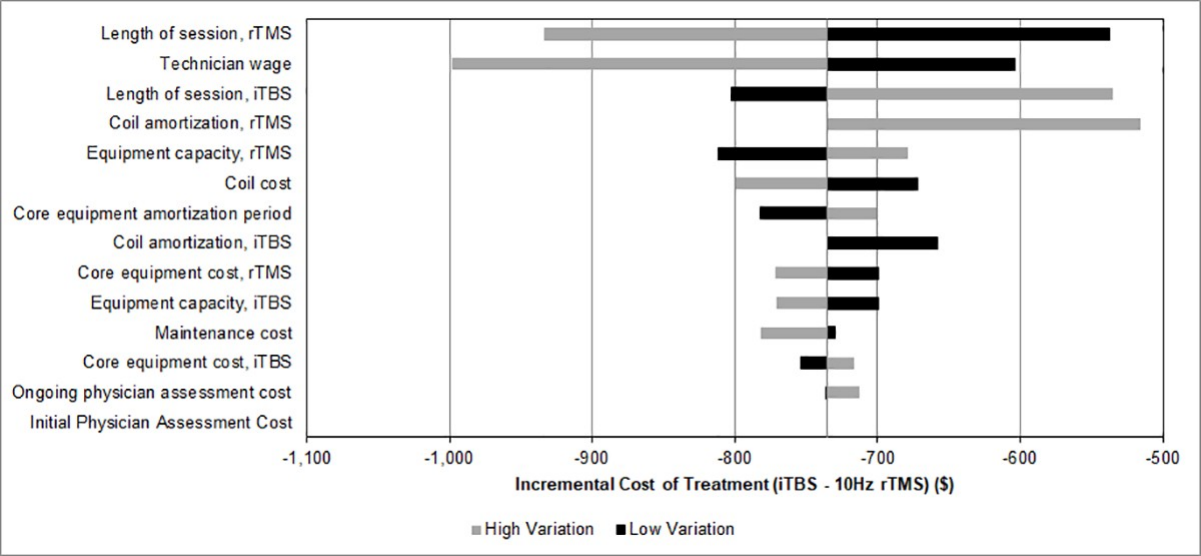
One-way sensitivity analyses on incremental cost of treatment per patient. High variation result reflects the result when the high end of the range for the specified parameter was used. Low variation reflects the result when the low end of the range for the specified parameter was used. iTBS indicates intermittent theta burst stimulation; rTMS, repetitive transcranial magnetic stimulation.

**Fig 2:**
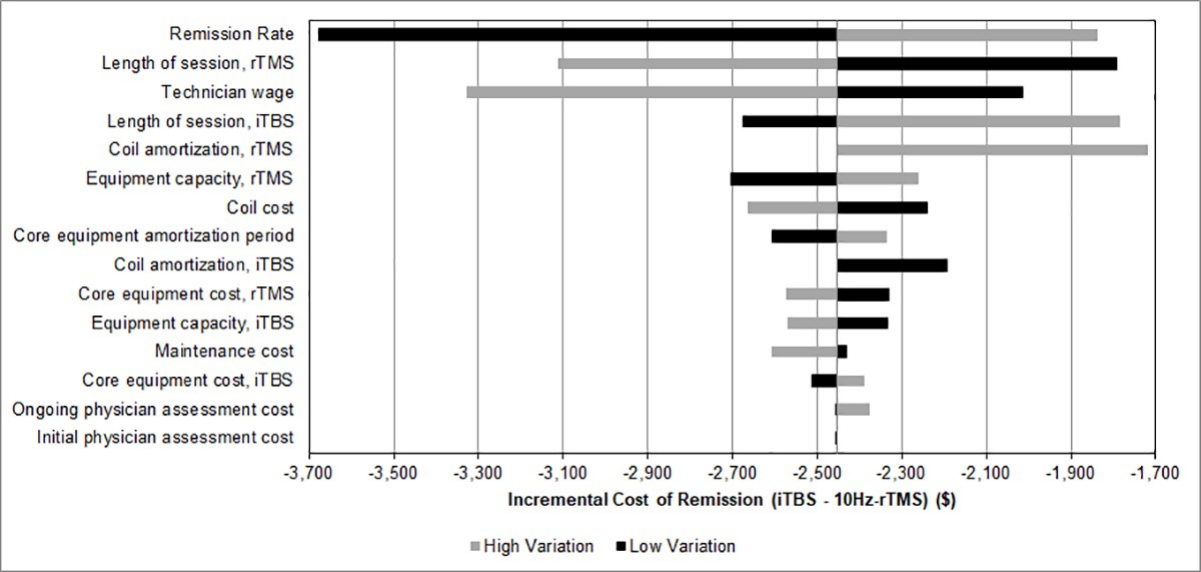
One-way sensitivity analyses results on incremental cost of remission. High variation result reflects the result when the high end of the range for the specified parameter was used. Low variation reflects the result when the low end of the range for the specified parameter was used. iTBS indicates intermittent theta burst stimulation; rTMS, repetitive transcranial magnetic stimulation.

One-way sensitivity analyses conducted on all variables in the study are presented in Figs 1 and 2. None of the tested scenarios resulted in iTBS being more expensive per course or per remission then 10Hz rTMS. 10Hz rTMS session length was associated with the largest variation in cost of treatment. When 10Hz rTMS length of session was varied, incremental cost ranged from a savings of $537 to $933 per course favouring iTBS over 10Hz rTMS. Varying the remission rate yielded the largest changes in estimates of cost per remission (Fig 2). When remission rate was varied over a range of 20% to 40%, the incremental cost per remission ranged from a savings of $1,839 to $3,677 per remission, favouring iTBS over 10Hz rTMS.

To ensure robustness of study results, patients’ prior history of ECT and number of rescheduled treatment sessions were also added as nuisance covariates into the regression model, as they were identified as being significantly different between treatment groups (*p* < 0.05). The inclusion of both variables had a minimal, not statistically significant effect on the incremental costs per course of treatment and per remission, and conclusions remained the same (S1 Table).

## Discussion

In the THREE-D trial, 3 min iTBS showed non-inferior outcomes to conventional 37.5 min 10Hz rTMS in reducing symptom severity for MDD patients.[21] In this follow-up cost-analysis based on the THREE-D trial outcome data, iTBS was found to be the less costly strategy from the healthcare system perspective, yielding an estimated savings of $735 (95% CI 688 to 783) per course of treatment delivered and of $2,451 (95% CI 2,293 to 2,610) per remission achieved. The lower cost of iTBS per course and per remission was largely attributable to the shorter technician time needed per session, the estimated increases in daily treatment capacity per device, and the lower amortized equipment costs per patient.

Currently in the US, rTMS is covered by federal and commercial healthcare insurers for the treatment of patients with MDD who have not achieved remission with conventional pharmacotherapy.[18,30] With the recent US FDA approval of iTBS, clinics can offer the benefits of rTMS treatment, with the added advantage of shorter treatment sessions.[26] Outside the US, the United Kingdom’s National Institute for Health and Care Excellence (NICE) has recommended rTMS for treatment of medication-resistant depression.[33] In Canada, although rTMS carries Health Canada approval for treatment of MDD, treatment is currently funded under the provincial health insurance plans only in Quebec and Saskatchewan.[17] Despite these differences in coverage, all of these organizations still emphasize the need for research to further optimize and inform decisions regarding the efficacy and cost-efficacy of rTMS compared to other available treatment strategies in MDD. While this study specifically assesses the direct treatment cost of iTBS, the results of this study provide quantitative evidence that iTBS may be a potentially affordable rTMS protocol for reimbursement.

Where previous studies have suggested that rTMS has the potential to be cost-effective when compared to ECT for TRD,[34] to our knowledge, this is the first study measuring the differences in treatment costs between two different protocols of rTMS treatment. In the US, published recommendations have suggested a cost ranging from $6,000 to $12,000 for an acute course of 20 to 30 rTMS sessions (i.e. $200 to $400 per session),[17,18] and current reimbursement for rTMS typically falls in the range of $120 to $250 per session among public and private coverage plans. Similarly, current costs in Canada fall in the range of $60 to $200 per session where publicly or privately funded rTMS is available. In Europe, the cost of rTMS in private clinics or large urban centres can fall in the range of $60 to $300 or higher per session. In all cases, the resultant per course costs present a significant economic obstacle to rTMS achieving meaningful reductions in the overall prevalence of MDD (~4.3% of the general population [1]) at a cost that is viable for the healthcare system as a whole. This study demonstrates that iTBS can achieve a several-fold reduction in cost per course delivered and a still larger reduction in the cost per remission achieved, without compromising therapeutic effectiveness. Such improvements may render use of rTMS more viable as a practical and less costly intervention for achieving meaningful, system-wide reductions in the prevalence and burden of disease associated with MDD.

Strength of the present analysis rests on its usage of outcome data from the THREE-D trial, the largest effectiveness trial of rTMS completed to date. The THREE-D trial was designed to be generalizable with inclusion and exclusion criteria that roughly corresponding with real-world clinical practice, as delineated in recent consensus rTMS recommendations.[18,21] Additional strengths include the testing of a range of assumptions in costs and remission rates, which bolster the robustness of the finding that iTBS offers several-fold improvements in cost per course and cost per remission across a range of possible real-world scenarios for equipment, personnel costs, and treatment outcomes.

At the same time, certain limitations warrant mention. As this study specifically considers the differences in the direct medical costs associated with delivering iTBS or 10Hz rTMS treatment, it does not consider indirect contributors to costs of running an rTMS clinic such as rent, utilities, facility fees, insurance, and electronic health record maintenance, all of which can vary greatly based on location. In addition, because this study only considered the direct costs associated with treatment, as over 30% of patients in both treatment groups in the THREE-D trial were currently employed, cost differences may have been underestimated by not considering the impact of shorter treatment sessions on patients’ absences from work and productivity losses.

There also are a range of possible estimates for parameters associated with equipment lifespan and equipment capacity that may have a large impact on the generalizability of this study’s results. To alleviate these concerns, expert opinions were sought to obtain conservative estimates of equipment capacity, lifespan, session duration and remission rate to avoid biasing cost measures associated with each intervention. Results from this study are relatively context specific, for example, by assuming a constant measure of equipment capacity, this study suggests that both treatments would see consistent core equipment usage over their lifespan. In practice, as responders may require repeated courses of treatment and there is already evidence of limited clinical capacity and increased wait times for conventional rTMS sessions,[12] this estimate may be conservative. It is also likely that the demand for rTMS may be higher or grow to support the expanding volume of patients with TRD. In addition, this study did not consider the cost of follow-up maintenance treatments that may be required to sustain remission, since at present, there is no consensus on what maintenance regimen (if any) should be applied among treatment responders.[35]

It is important to note that given the shorter duration of iTBS sessions, this study did not assess the impact of repeating treatment sessions per day, which has been recently explored as a strategy for potentially accelerating courses of treatment from weeks to days.[36] Clinical evidence has not yet determined if any difference in the efficacy of treatment would also be impacted.[36] The question of cost-savings associated with short courses with multiple daily sessions thus awaits future studying using robust outcome data from large trials, once these become available.

## Conclusions

In summary, this study demonstrates the potential cost of iTBS treatment for patients with TRD when compared to 10Hz rTMS. Where other published studies have suggested the notion of shorter rTMS protocols potentially increasing treatment capacity and therefore permitting lower treatment costs and wider affordability, this study evaluates the potential cost-savings between equally effective rTMS treatment protocols.[37] Although iTBS equipment is more expensive when compared to 10Hz rTMS, the impact of a shorter session duration on technician time and treatment capacity has the potential to result in cost-savings per patient and per remission. These cost savings are several-fold in magnitude and may be sufficient to position rTMS as a more economically viable intervention for achieving meaningful reductions in the system-wide prevalence and burden of disease for MDD in the general population, in future years.

## Supporting information

S1 Table Incremental per patient costs and non-parametric interval estimates. Note: Costs are in 2018 United States dollars (USD) and rounded to the nearest dollar. *Estimated using a generalized linear regression model with treatment type, history of ECT and the number of rescheduled treatment sessions as the independent variables. Non-parametric bootstrapping was conducted using 1,000 replications to generate uncertainty intervals. Abbreviations: iTBS, intermittent theta burst stimulation; rTMS, repetitive transcranial magnetic stimulation; SD, standard deviation.
(DOCX)

## References

[1] World Health Organization. Global burden of mental disorders and the need for a comprehensive, coordinated response from health and social sectors at the country level. Report by the Secretariat. Geneva, Switzerland: 2011.

[2] Friedrich MJ. Depression is the leading cause of disability around the world. JAMA 2017;317(15):1517–1517. 10.1001/jama.2017.3826 28418490

[3] Baldwin RC, Simpson S. Treatment resistant depression in the elderly: a review of its conceptualisation, management and relationship to organic brain disease. Journal of Affective Disorders 1997;46(3):163–73. 10.1016/s0165-0327(97)00143-2 9547114

[4] Souery D, Amsterdam J, de Montigny C, Lecrubier Y, Montgomery S, Lipp O, et al Treatment resistant depression: methodological overview and operational criteria. European Neuropsychopharmacology 1999;9(1):83–91. 10082232 10.1016/s0924-977x(98)00004-2

[5] Rush AJ, Kraemer HC, Sackeim HA, Fava M, Trivedi MH, Frank E, et al Report by the ACNP Task Force on Response and Remission in Major Depressive Disorder. Neuropsychopharmacology 2006;31(9):1841–53. 10.1038/sj.npp.1301131 16794566

[6] Rush AJ, Trivedi MH, Wisniewski SR, Nierenberg AA, Stewart JW, Warden D, et al Acute and Longer-Term Outcomes in Depressed Outpatients Requiring One or Several Treatment Steps: A STAR*D Report. AJP 2006;163(11):1905–17. 10.1176/ajp.2006.163.11.1905 17074942

[7] Rizvi SJ, Grima E, Tan M, Rotzinger S, Lin P, McIntyre RS, et al Treatment-resistant depression in primary care across Canada. The Canadian Journal of Psychiatry 2014;59(7):349–57. 10.1177/070674371405900702 25007419 PMC4086317

[8] Nemeroff CB. Prevalence and management of treatment-resistant depression. Journal of Clinical Psychiatry 2007;68(8):17. 17640154

[9] Malhi GS, Parker GB, Crawford J, Wilhelm K, Mitchell PB. Treatment-resistant depression: resistant to definition? Acta Psychiatrica Scandinavica 2005;112(4):302–9. 10.1111/j.1600-0447.2005.00602.x 16156838

[10] Olchanski N, McInnis Myers M, Halseth M, Cyr PL, Bockstedt L, Goss TF, et al The Economic Burden of Treatment-Resistant Depression. Clinical Therapeutics 2013;35(4):512–22. 10.1016/j.clinthera.2012.09.001 23490291

[11] Lisanby SH. Electroconvulsive Therapy for Depression. N Engl J Med 2007;357(19):1939–45. 10.1056/NEJMct075234 17989386

[12] Downar J, Blumberger DM, Daskalakis ZJ. Repetitive transcranial magnetic stimulation: an emerging treatment for medication-resistant depression. Canadian Medical Association Journal 2016;188(16):1175–7. 10.1503/cmaj.151316 27551033 PMC5088079

[13] Getty SS, Faziola LR. Adverse effects of electroconvulsive therapy on cognitive performance. Ment Illn 2017;9(2). 10.4081/mi.2017.7181 PMC5661121 29142661

[14] George MS, Wassermann EM, Williams WA, Callahan A, Ketter TA, Basser P, et al Daily repetitive transcranial magnetic stimulation (rTMS) improves mood in depression. Neuroreport: An International Journal for the Rapid Communication of Research in Neuroscience 1995; 6(14): 1853–6. 10.1097/00001756-199510020-00008 8547583

[15] Pascual-Leone A, Rubio B, Pallardó F, Catalá MD. Rapid-rate transcranial magnetic stimulation of left dorsolateral prefrontal cortex in drug-resistant depression. The Lancet 1996;348(9022):233–7. 10.1016/s0140-6736(96)01219-6 8684201

[16] Brunoni AR, Chaimani A, Moffa AH, Razza LB, Gattaz WF, Daskalakis ZJ, et al Repetitive Transcranial Magnetic Stimulation for the Acute Treatment of Major Depressive Episodes: A Systematic Review With Network Meta-analysis. JAMA Psychiatry 2017;74(2):143–52. 10.1001/jamapsychiatry.2016.3644 28030740

[17] Health Quality Ontario. Repetitive transcranial magnetic stimulation for treatment-resistant depression: an economic analysis. Ont Health Technol Assess Ser. 2016 3;16(6):1–51. Available from: http://www.hqontario.ca/evidence/publications-and-ohtacrecommendations/ontario-health-technology-assessment-series/econ-rtms 27110317 PMC4808718

[18] McClintock SM, Reti IM, Carpenter LL, McDonald WM, Dubin M, Taylor SF, et al Consensus recommendations for the clinical application of repetitive transcranial magnetic stimulation (rTMS) in the treatment of depression. The Journal of clinical psychiatry 2018;79(1). 10.4088/JCP.16cs10905 PMC5846193 28541649

[19] O’Reardon JP, Solvason HB, Janicak PG, Sampson S, Isenberg KE, Nahas Z, et al Efficacy and safety of transcranial magnetic stimulation in the acute treatment of major depression: a multisite randomized controlled trial. Biological psychiatry 2007;62(11):1208–16. 10.1016/j.biopsych.2007.01.018 17573044

[20] George MS, Lisanby SH, Avery D, McDonald WM, Durkalski V, Pavlicova M, et al Daily left prefrontal transcranial magnetic stimulation therapy for major depressive disorder: a sham-controlled randomized trial. Archives of general psychiatry 2010;67(5):507–16. 10.1001/archgenpsychiatry.2010.46 20439832

[21] Blumberger DM, Vila-Rodriguez F, Thorpe KE, Feffer K, Noda Y, Giacobbe P, et al Effectiveness of theta burst versus high-frequency repetitive transcranial magnetic stimulation in patients with depression (THREE-D): a randomised non-inferiority trial. The Lancet 2018;391(10131):1683–92. 10.1016/S0140-6736(18)30295-2 29726344

[22] Huang Y-Z, Edwards MJ, Rounis E, Bhatia KP, Rothwell JC. Theta Burst Stimulation of the Human Motor Cortex. Neuron 2005;45(2):201–6. 10.1016/j.neuron.2004.12.033 15664172

[23] Di Lazzaro V, Dileone M, Pilato F, Capone F, Musumeci G, Ranieri F, et al Modulation of motor cortex neuronal networks by rTMS: comparison of local and remote effects of six different protocols of stimulation. Journal of neurophysiology 2011;105(5):2150–6. 10.1152/jn.00781.2010 21346213

[24] Li CT, Chen MH, Juan CH, Huang HH, Chen LF, Hsieh JC, et al Efficacy of prefrontal theta-burst stimulation in refractory depression: a randomized sham-controlled study. Brain 2014;137(7):2088–98. 24817188 10.1093/brain/awu109

[25] Plewnia C, Pasqualetti P, Grobe S, Schlipf S, Wasserka B, Zwissler B, et al Treatment of major depression with bilateral theta burst stimulation: a randomized controlled pilot trial. Journal of affective disorders 2014;156:219–23. 10.1016/j.jad.2013.12.025 24411682

[26] Brooks M. FDA Clears 3-Minute Brain Stimulation Protocol for Depression. MedScape Medical News Published Online First: 22 8 2018 Available from: https://www.medscape.com/viewarticle/901052 Cited 31 August 2018.

[27] U.S. Centers for Medicare and Medicaid Services. Physician fee schedule search. 2018. Available from: https://www.cms.gov/apps/physician-fee-schedule/search/search-criteria.aspx Cited 9 August 2018.

[28] Canadian Agency for Drugs and Technology in Health. Diagnostic imaging equipment replacement and upgrade in Canada (Environmental scan; no.56). Ottawa, Ontario: 2016. Available from: https://www.cadth.ca/diagnostic-imaging-equipment-replacement-and-upgrade Cited 10 August 2018.

[29] Oberman L, Edwards D, Eldaief M, Pascual-Leone A. Safety of theta burst transcranial magnetic stimulation: a systematic review of the literature. Journal of Clinical Neurophysiology 2011;28(1):67 10.1097/WNP.0b013e318205135f 21221011 PMC3260517

[30] Gaynes BN, Lloyd SW, Lux L, Gartlehner G, Hansen RA, Brode S, et al Repetitive transcranial magnetic stimulation for treatment-resistant depression: a systematic review and meta-analysis. J Clin Psychiatry 2014; 75(5):477–89. 10.4088/JCP.13r08815 24922485

[31] George MS, Taylor JJ, Short EB. The expanding evidence base for rTMS treatment of depression. Current opinion in psychiatry 2013;26(1):13 10.1097/YCO.0b013e32835ab46d 23154644 PMC4214363

[32] Glick HA, Doshi JA, Sonnad SS, Polsky D. Economic evaluation in clinical trials. OUP Oxford; 2014.

[33] National Institute for Health and Care Excellence. Repetitive transcranial magnetic stimulation for depression. London, UK: 2015. Available from: https://www.nice.org.uk/guidance/ipg542/resources/repetitive-transcranial-magnetic-stimulation-for-depression-pdf-1899871923433669 Cited 13 August 2018.

[34] Vallejo-Torres L, Castilla I, González N, Hunter R, Serrano-Pérez P, Perestelo-Pérez L. Cost-effectiveness of electroconvulsive therapy compared to repetitive transcranial magnetic stimulation for treatment-resistant severe depression: a decision model. Psychological medicine 2015;45(7):1459–70. 10.1017/S0033291714002554 25354790 PMC4413854

[35] Milev RV, Giacobbe P, Kennedy SH, Blumberger DM, Daskalakis ZJ, Downar J, et al Canadian Network for Mood and Anxiety Treatments (CANMAT) 2016 Clinical Guidelines for the Management of Adults with Major Depressive Disorder: Section 4. Neurostimulation Treatments. Canadian journal of psychiatry 2016;61(9):561–75. 10.1177/0706743716660033 27486154 PMC4994792

[36] Duprat R, Desmyter S, van Heeringen K, Van den Abbeele D, Tandt H, Bakic J, et al Accelerated intermittent theta burst stimulation treatment in medication-resistant major depression: a fast road to remission? Journal of affective disorders 2016;200:6–14. 10.1016/j.jad.2016.04.015 27107779

[37] Bakker N, Shahab S, Giacobbe P, Blumberger DM, Daskalakis ZJ, Kennedy SH, Downar J. rTMS of the dorsomedial prefrontal cortex for major depression: safety, tolerability, effectiveness, and outcome predictors for 10 Hz versus intermittent theta-burst stimulation. Brain stimulation 2015;8(2):208–15. 10.1016/j.brs.2014.11.002 25465290

