# Supplementary material for: Implementation of intermittent theta burst stimulation compared to conventional repetitive transcranial magnetic stimulation in patients with treatment resistant depression: A cost analysis
Source: PLoS One. 2019 Sep 12;14(9):e0222546. doi: 10.1371/journal.pone.0222546 (PMC6742475; doi:10.1371/journal.pone.0222546)
Supplement: S1 Table — Note: Costs are in 2018 United States dollars (USD) and rounded to the nearest dollar. *Estimated using a generalized linear regression model with treatment type, history of ECT and the number of rescheduled treatment sessions as the independent variables. Non-parametric bootstrapping was conducted using 1,000 replications to generate uncertainty intervals. Abbreviations: iTBS, intermittent theta burst stimulation; rTMS, repetitive transcranial magnetic stimulation; SD, standard deviation. (DOCX) [file pone.0222546.s001.docx]

**SUPPORTING INFORMATION**

|  | **Incremental Cost (iTBS – 10Hz rTMS)** | | |
| --- | --- | --- | --- |
| **Parameter** | **Mean (SD)** | **95% Confidence Interval^*^** | **P Value^*^** |
| Cost of treatment | -724 (25) | -774 – -674 | P < 0.001 |
| Cost of remission | -2,413 (85) | -2,579 – -2,247 | P < 0.001 |

**S1 Table. Incremental per patient costs and non-parametric interval estimates**

Note: Costs are in 2018 United States dollars (USD) and rounded to the nearest dollar.

*Estimated using a generalized linear model with treatment type, history of ECT and the number of rescheduled treatment sessions as the independent variables. Non-parametric bootstrapping was conducted using 1,000 replications to generate uncertainty intervals.

Abbreviations: iTBS, intermittent theta burst stimulation; rTMS, repetitive transcranial magnetic stimulation; SD, standard deviation.
